# Annotated Sequence of *Mycobacterium tuberculosis* MTBR3/09 Isolated from a Sputum Sample in Malaysia

**DOI:** 10.1128/genomeA.00517-16

**Published:** 2016-06-23

**Authors:** Rahizan Issa, Valentinus H. Seradja, Mohd Khairul Hafizi Abdullah, Hatijah Abdul

**Affiliations:** Bacteriology Unit, Infectious Disease Research Centre, Institute for Medical Research, Kuala Lumpur, Malaysia

## Abstract

This is a report of the annotated genome sequence of *Mycobacterium tuberculosis* MTBR3/09. The organism was isolated from a sputum sample in Malaysia.

## GENOME ANNOUNCEMENT

In Malaysia, a report states that the rate of progress against tuberculosis (TB) has increased each year between 2011 and 2014 (1). At these present rates of progress, the target of TB elimination by 2035 seems remote (2). In this report, the clinical isolate *M. tuberculosis* MTBR3/09 from a male patient was subjected to whole-genome shotgun sequencing.

The total genomic DNA of *M. tuberculosis* MTBR3/09 was extracted using QIAxtractor and reagent packs (Qiagen, Düsseldorf, Germany), according to the manufacturer’s instruction. DNA concentrations were measured using a NanoVue Plus (GE Healthcare, Buckinghamshire, United Kingdom) spectrophotometer. The Illumina MiSeq platform was used for whole-genome shotgun sequencing.

This resulted in 9,073,418 filtered reads, with an average read length of 109.0 bp and approximately 248-fold coverage. The filtered reads were *de novo* assembled with CLC Genomics Workbench version 6.0.1, generating 167 contigs (*N*_50_, 82,794 bp). The genome contains a total of 4,349,175 bp, with a G+C content of 65.59%.

The NCBI Prokaryotic Genome Annotation Pipeline was used. The genome was identified as having 4,128 genes, 3,950 coding sequences (CDSs), and 129 pseudogenes. Three types of rRNA (5S, 16S, and 23S) were annotated, and each had one rRNA. One clustered regularly interspaced short palindromic repeat (CRISPR) array, one noncoding RNA (ncRNA), and 45 tRNAs were annotated in this genome.

The SpolPred software was used to determine the spoligotype of *M. tuberculosis* MTBR3/09. It was determined to be 000000000003731, belonging to East Asian (Beijing) lineage.

### Nucleotide sequence accession number.

This whole-genome shotgun project has been deposited in DDBJ/ENA/GenBank under the accession no. LATP00000000. The version described in this paper is the first version.

## References

[1] The World Bank 2016 Incidence of tuberculosis (per 100,000 people). The World Bank, Washington, DC http://data.worldbank.org/indicator/SH.TBS.INCD.

[2] Zumla A, George A, Sharma V, Herbert RHN, Oxley A, Oliver M 2014 The WHO global tuberculosis report–further to go. Lancet Glob Health 3:e10–e12. 25539957 10.1016/S2214-109X(14)70361-4

